# Associations between blood manganese levels and sarcopenia in adults: insights from the National Health and Nutrition Examination Survey

**DOI:** 10.3389/fpubh.2024.1351479

**Published:** 2024-05-13

**Authors:** Bing Xu, Zuo-xi Chen, Wu-jie Zhou, Jia Su, Qiang Zhou

**Affiliations:** Department of Orthopedic Surgery, Wenzhou Hospital of Integrated Traditional Chinese and Western Medicine, Wenzhou, Zhejiang, China

**Keywords:** blood manganese, sarcopenia, adolescents, cross-sectional studies, National Health and Nutrition Examination Survey

## Abstract

**Background:**

While increasing concerns arise about the health effects of environmental pollutants, the relationship between blood manganese (Mn) and sarcopenia has yet to be fully explored in the general population.

**Objective:**

This study aims to investigate the association between blood manganese (Mn) levels and sarcopenia in adults.

**Methods:**

In our study, we evaluated 8,135 individuals aged 18–59 years, utilizing data from the National Health and Nutrition Examination Survey (NHANES) spanning 2011 to 2018. We employed generalized additive model (GAM) to discern potential non-linear relationships and utilized the two-piecewise linear regression model to probe the association between blood Mn levels and sarcopenia.

**Results:**

After adjusting for potential confounders, we identified non-linear association between blood Mn levels and sarcopenia, with an inflection point at 13.45 μg/L. The effect sizes and the confidence intervals on the left and right sides of the inflection point were 1.006 (0.996 to 1.048) and 1.082 (1.043 to 1.122), respectively. Subgroup analysis showed that the effect sizes of blood Mn on sarcopenia have significant differences in gender and different BMI groups.

**Conclusion:**

Our results showed that a reverse U-shaped curve between blood Mn levels and sarcopenia, with an identified the inflection point at blood Mn level of 13.45 μg/L.

## Introduction

Sarcopenia, characterized by an age-related decrease in skeletal muscle mass and function, is a recognized risk factor for significant adverse health outcomes such as frailty, disability, institutionalization, and mortality (1–3). Starting at age 30, there is a yearly loss of 0.5–1% in skeletal muscle mass, and this decline becomes markedly faster after the age of 65 (4). Sarcopenia affects over 40% of individuals aged 70 and above, equating to nearly 50 million people globally. Given the recent rise in life expectancy, this figure is projected to multiply tenfold by 2050. It is worth noting that while sarcopenia is predominantly diagnosed in the older person, it can manifest in various clinical scenarios (5).

Manganese (Mn) is an essential trace element that is involved in numerous physiological activities, including protein synthesis, signal transduction, and metabolism (6). The typical serum levels range from 4 to 15 μg/L in adults (7). It is a crucial component of enzymes that defend cells from oxidative stress, especially vital for the protection of brain and muscle cells that underpin neuromotor function (8, 9). However, consuming amounts that exceed the body’s homeostatic capacity can lead to toxic effects (8, 10). The main source of exposure to the general population is through the diet, with this element being present in many foods, including nuts, legumes, seeds, tea, whole grains, and leafy green vegetables (11). Dietary intake alone can surpass the body’s homeostatic capacity for Mn, it is generally uncommon for diet alone to result in Mn toxicity. However, certain conditions and lifestyles can disrupt this balance, potentially leading to excess accumulation. Exposure to Mn-containing dust or fumes in occupations such as mining, steel manufacturing, or welding can contribute to this imbalance (11). Additionally, the overuse of certain psychotropic drugs, such as ephedra, can also lead to excess Mn accumulation (10). Notably, Mn toxicity has been associated with neurological disorders (12), but its impact on musculoskeletal health, particularly sarcopenia, is not well understood.

However, no study has yet investigated potential muscle deficits in relation to blood Mn in the general adult population. As such, our study draws on data from participants aged 18–59 across the National Health and Nutrition Examination Survey (NHANES) 2011–2018 in order to evaluate the relationship between the concentration of blood Mn and sarcopenia.

## Methods

### Study design

Continuous variables were expressed as mean ± standard deviation or median, and categorical variables were expressed in frequency or as a percentage. The One-Way ANOVA, Kruskal-Wallis H test, and chi-square tests were utilized to determine statistical differences in the means and proportions across groups. A univariate linear regression model assessed the associations between blood Mn levels and sarcopenia. Both non-adjusted and multivariate-adjusted models were reported. Following the STROBE statement guidelines, we concurrently presented results from unadjusted, minimally adjusted, and fully adjusted analyses Covariance adjustments were made based on a principle that inclusion in the model altered the matched odds ratio by at least 10%. Furthermore, we employed a generalized additive model (GAM) to detect non-linear relationships. Upon observing a non-linear correlation, a two-piecewise linear regression model was implemented to estimate the threshold effect of blood Mn concentration on sarcopenia, illustrated by the smoothing plot. Subgroup analyses were conducted using stratified linear regression models, with subgroup modifications and interactions being examined via the likelihood ratio test.

### Study population

The National Health and Nutrition Examination Survey (NHANES) is a population-based cross-sectional survey administered by the National Center for Health Statistics (NCHS) with the objective of evaluating the health and nutrition status of both adults and children in the United States. Employing a sophisticated multistage sampling design, the NHANES collects data from a representative subset of the population. Since its inception in the 1960s, the NHANES has been conducted on a regular basis, furnishing invaluable insights into the health and nutrition of the American population. Data from the official NHANES website, spanning the period from 2011 to March 2018, were accessed. Additional details can be found therein.

### Definition of sarcopenia and assessment methodology

Sarcopenia, defined as an age-related decline in skeletal muscle mass and function, poses significant risks for adverse health outcomes like frailty and disability. In our study, sarcopenia was assessed using Dual-Energy X-ray Absorptiometry (DXA) whole body evaluations, performed with Hologic Discovery Model A densitometers. These evaluations primarily focused on measuring the appendicular skeletal muscle mass to accurately quantify muscle content. The DXA scan exclusions included pregnancy, a weight surpassing 300 pounds (136 kg) due to the scanner’s weight capacity, height exceeding 6′5″ given the DXA table’s limitations, recent barium-based radiographic contrast exposure within the past 7 days, or engagement in nuclear medicine studies in the prior 3 days. All scans were orchestrated using the Hologic software (V.8.26: a3), with DXA being instrumental in gauging the appendicular skeletal muscle mass (13). The sarcopenia index, used to diagnose sarcopenia, was calculated by dividing the total appendicular skeletal muscle mass (in kilograms) by the body mass index (BMI, kg/m^2). For diagnosing sarcopenia in this study, we employed sex-specific cut-off values for the sarcopenia index, setting them at 0.789 for men and 0.512 for women, as recommended by the National Institutes of Health (FNIH) guidelines (14).

### Assessment of blood Mn

Following a basic dilution sample preparation, the Mn concentration in whole blood specimens was determined using mass spectrometry. The Mn exhibited a lower limit of detection (LOD) at an estimated 0.99 μg/L. Values measured beneath the detection limit were denoted as the LOD over the square root of 2. For an in-depth overview, refer to the NHANES website (15).

### Covariates

Demographic and lifestyle factors are assessed using questionnaires. The baseline data include age (younger than 40, 40 and older), gender (male, female), educational levels (less than high school education, some high school, high school graduate/GED, some college or associate’s degree, college graduate or higher), alcohol intake (non-drinker, 1 to less than 5 drinks per month, 5 to less than 10 drinks per month, or 10 or more drinks per month), smoking status (current, former, or never smoker), physical exercises (none, moderate, vigorous), and poverty level index (PLI). Diabetes is defined as having a fasting glucose level of 126 mg/dL or higher or reporting a previous diagnosis. Hypertension is defined as having persistent resting blood pressure (BP) at or above 140/90 mmHg or reporting a previous diagnosis. Cardiovascular disease encompass self-reported medical diagnoses, including congestive heart failure, angina, myocardial infarction, and stroke.

### Statistical analysis

Statistical evaluations were executed using the R statistical software^1^ and Empower Stats (X&Y Solutions, Inc., Boston, MA).^2^ Variables adhering to a normal distribution were articulated as mean ± standard deviation (SD). A two-sided *p*-value of <0.01 was deemed indicative of statistical significance.

## Results

### Baseline characteristics of the study participants

Of the 8,135 participants in the 2011–2018 NHANES study, 667 were diagnosed with sarcopenia. Among the participants diagnosed with sarcopenia, which constituted 8.2% of our study population, the average age was 43.05 ± 12.23 years. Approximately 62.82% of the participants were aged 40 years or above. In contrast, individuals younger than 40 years comprised 37.18% of the study population. Specifically, younger participants (under 40) exhibited an average Mn level of 10.37 μg/L, while their older counterparts exhibited an average level of 10.15 μg/L. Table 1 compares the baseline demographic, clinical, and biochemical characteristics of individuals by tertiles of the Mn concentration groups. No significant differences were observed in age, diabetes prevalence, cardiovascular disease incidence, and the poverty index across the various blood Mn concentration groups. The mean age of the cohort was 37.354 ± 12.369 years, and 49.81% of the participants were male. Compared to subjects in the lowest tertile of the blood Mn concentration group, those in the highest tertile exhibited the following characteristics: younger age, higher proportion of females, increased waist circumference, higher proportion of 10+ drinks per month, higher proportion of current smokers, lower proportion individuals diagnosed with hypertension proportion of individuals with a college higher, discernible decline in engagement in vigorous physical activities, and lower proportion of individuals with BMI <25.

**Table 1:** Baseline characteristics of participants.

Blood Mn	Q1 (1.88–7.65)	Q2 (7.65–9.54)	Q3 (9.54–12.05)	Q4 (12.05–52.01)	*p* value
Age					0.126
<=40	1,113 (55.045%)	1,175 (57.542%)	1,174 (57.634%)	1,192 (58.604%)	
>40	909 (44.955%)	867 (42.458%)	863 (42.366%)	842 (41.396%)	
Gender					<0.001
Male	1,268 (62.710%)	1,129 (55.289%)	986 (48.405%)	669 (32.891%)	
Female	754 (37.290%)	913 (44.711%)	1,051 (51.595%)	1,365 (67.109%)	
Waist circumference	95.704 ± 16.304	96.357 ± 16.686	96.650 ± 16.204	94.995 ± 16.488	0.007
Alcohol consumption					<0.001
Non-drinker	924 (45.697%)	1,026 (50.245%)	990 (48.601%)	960 (47.198%)	
1 to <5 drinks/month	457 (22.601%)	388 (19.001%)	318 (15.611%)	315 (15.487%)	
5 to <10 drinks/month	308 (15.232%)	269 (13.173%)	293 (14.384%)	250 (12.291%)	
10+ drinks/month	333 (16.469%)	359 (17.581%)	436 (21.404%)	509 (25.025%)	
Smoking status					<0.001
Current	1,136 (56.182%)	1,217 (59.598%)	1,310 (64.310%)	1,402 (68.928%)	
Former	358 (17.705%)	352 (17.238%)	339 (16.642%)	310 (15.241%)	
Never smoker	528 (26.113%)	473 (23.164%)	388 (19.048%)	322 (15.831%)	
Diabetes					0.282
Yes	150 (7.418%)	126 (6.170%)	153 (7.511%)	136 (6.686%)	
No	1872 (92.582%)	1916 (93.830%)	1884 (92.489%)	1898 (93.314%)	
Hypertension					0.001
Yes	494 (24.431%)	443 (21.694%)	431 (21.159%)	395 (19.420%)	
No	1,528 (75.569%)	1,599 (78.306%)	1,606 (78.841%)	1,639 (80.580%)	
Cardiovascular disease					0.779
Yes	24 (1.187%)	20 (0.979%)	19 (0.933%)	18 (0.885%)	
No	1998 (98.813%)	2022 (99.021%)	2018 (99.067%)	2016 (99.115%)	
Educational levels					<0.001
Less than high school education	91 (4.500%)	111 (5.436%)	115 (5.646%)	160 (7.866%)	
Less than high school education	243 (12.018%)	222 (10.872%)	250 (12.273%)	222 (10.914%)	
High school graduate/GED	502 (24.827%)	466 (22.821%)	422 (20.717%)	451 (22.173%)	
Some college or associate’s degree	732 (36.202%)	731 (35.798%)	692 (33.972%)	632 (31.072%)	
College graduate or higher	454 (22.453%)	512 (25.073%)	558 (27.393%)	569 (27.974%)	
Physical activity					<0.001
Vigorous	564 (27.893%)	489 (23.947%)	484 (23.760%)	387 (19.027%)	
Moderate	414 (20.475%)	466 (22.821%)	434 (21.306%)	449 (22.075%)	
None	1,044 (51.632%)	1,087 (53.232%)	1,119 (54.934%)	1,198 (58.899%)	
BMI					0.019
<=25	690 (34.125%)	672 (32.909%)	653 (32.057%)	725 (35.644%)	
>25, <=30	675 (33.383%)	625 (30.607%)	644 (31.615%)	605 (29.744%)	
>30	657 (32.493%)	745 (36.484%)	740 (36.328%)	704 (34.612%)	
Poverty level index	2.459 ± 1.631	2.453 ± 1.647	2.419 ± 1.604	2.394 ± 1.623	0.545

### Univariate analysis

The results of the univariate analysis are presented in Table 2. The results showed that age, BMI, waist circumference, hypertension, diabetes, smoking status, alcohol consumption, family PIR, Mn, and cardiovascular disease were positively correlated with sarcopenia. We also found that gender and physical activity were not associated with sarcopenia.

**Table 2:** The results of univariate analysis.

Variables	Statistics	Effect size (β)	*p* value
Age			
<=40	3,661 (45.003%)	1.0	
>40	4,474 (54.997%)	0.454 (0.386, 0.535)	<0.001
Gender			
Male	4,052 (49.809%)	1.0	
Female	4,083 (50.191%)	0.950 (0.811, 1.114)	0.529
Waist circumference	95.928 ± 16.431	1.047 (1.042, 1.051)	<0.001
Alcohol consumption			
Non-drinker	3,900 (47.941%)	1.0	
1 to <5 drinks/month	1,478 (18.168%)	0.805 (0.637, 1.019)	0.071
5 to <10 drinks/month	1,120 (13.768%)	0.943 (0.736, 1.209)	0.644
10+ drinks/month	1,637 (20.123%)	1.288 (1.058, 1.569)	0.011
Smoking status			
Current	5,065 (62.262%)	1.0	
Former	1,359 (16.706%)	1.085 (0.880, 1.338)	0.445
Never smoker	1711 (21.033%)	0.771 (0.623, 0.956)	0.017
Diabetes			
Yes	565 (6.945%)	1.0	
No	7,570 (93.055%)	0.356 (0.283, 0.448)	<0.001
Hypertension			
Yes	1,763 (21.672%)	1.0	
No	6,372 (78.328%)	0.576 (0.484, 0.684)	<0.001
Cardiovascular disease			
Yes	81 (0.996%)	1.0	
No	8,054 (99.004%)	0.357 (0.206, 0.621)	<0.001
Educational levels			
Less than high school education	477 (5.864%)	1.0	
Less than high school education	937 (11.518%)	0.381 (0.282, 0.513)	<0.001
High school graduate/GED	1841 (22.631%)	0.349 (0.268, 0.453)	<0.001
Some college or associate’s degree	2,787 (34.259%)	0.218 (0.168, 0.284)	<0.001
College graduate or higher	2093 (25.728%)	0.183 (0.137, 0.244)	<0.001
Physical activity			
Vigorous	1,924 (23.651%)	1.0	
Moderate	1,763 (21.672%)	0.891 (0.697, 1.138)	0.355
None	4,448 (54.677%)	1.106 (0.910, 1.345)	0.311
BMI			
<=25	2,740 (33.682%)	1.0	
>25, <=30	2,549 (31.334%)	2.777 (2.058, 3.749)	<0.001
>30	2,846 (34.985%)	8.134 (6.202, 10.667)	<0.001
Poverty level index	2.431 ± 1.626	0.894 (0.849, 0.940)	<0.001
Mn	10.269 ± 3.896	1.0389 (1.020,1.057)	<0.001

### The results of the relationship between blood Mn and sarcopenia

We applied a univariate linear regression model to assess the relationship between blood Mn levels and sarcopenia. Detailed results of the unadjusted and adjusted analyses are presented in Table 3. In the crude model, blood Mn exhibited positive correlation with sarcopenia (*β* = 1.039, 95% CI: 1.020 to 1.058, *p* < 0.001). In the model minimally adjusted for age and gender, the findings remained largely unaltered (*β* = 1.042, 95%CI: 1.024 to 1.061, *p* < 0.001). Likewise, in the model fully adjusted for additional covariates, the outcomes persisted in their consistency (*β* = 1.039, 95%CI: 1.019 to 1.060, *p* < 0.001). For the purpose of sensitivity analysis, we also handled blood Mn Categorical variable (Quartile), and found that the same trend was observed as well (p for trend was <0.001).

**Table 3:** Relationship between blood Mn and sarcopenia in different models.

Variable	Crude model (β, 95%CI, P)	Minimally adjusted model (β, 95%CI, P)	Fully adjusted model (β, 95%CI, P)
Blood Mn	1.039 (1.020, 1.058) 0.00003	1.042 (1.024, 1.061) <0.00001	1.039 (1.019, 1.060) 0.00012
Blood Mn (quartile)			
Q1	Ref	Ref	Ref
Q2	1.061 (0.831, 1.356) 0.63301	1.084 (0.848, 1.387) 0.51948	1.022 (0.790, 1.323) 0.86857
Q3	1.368 (1.084, 1.728) 0.00844	1.403 (1.110, 1.774) 0.00469	1.302 (1.017, 1.668) 0.03637
Q4	1.661 (1.325, 2.083) 0.00001	1.722 (1.372, 2.162) <0.00001	1.662 (1.306, 2.115) 0.00004
P for trend	<0.001	<0.001	<0.001

Crude model: we did not adjust other covariants. Minimally adjusted model: we adjusted age and gender. Fully adjusted model: we adjusted age, gender waist circumference, alcohol consumption, smoking status, diabetes, hypertension, cardiovascular, educational level, BMI, poverty level index. CI confidence interval, Ref reference.

### The results of relationship between non-linear relationship

**Figure 1:**
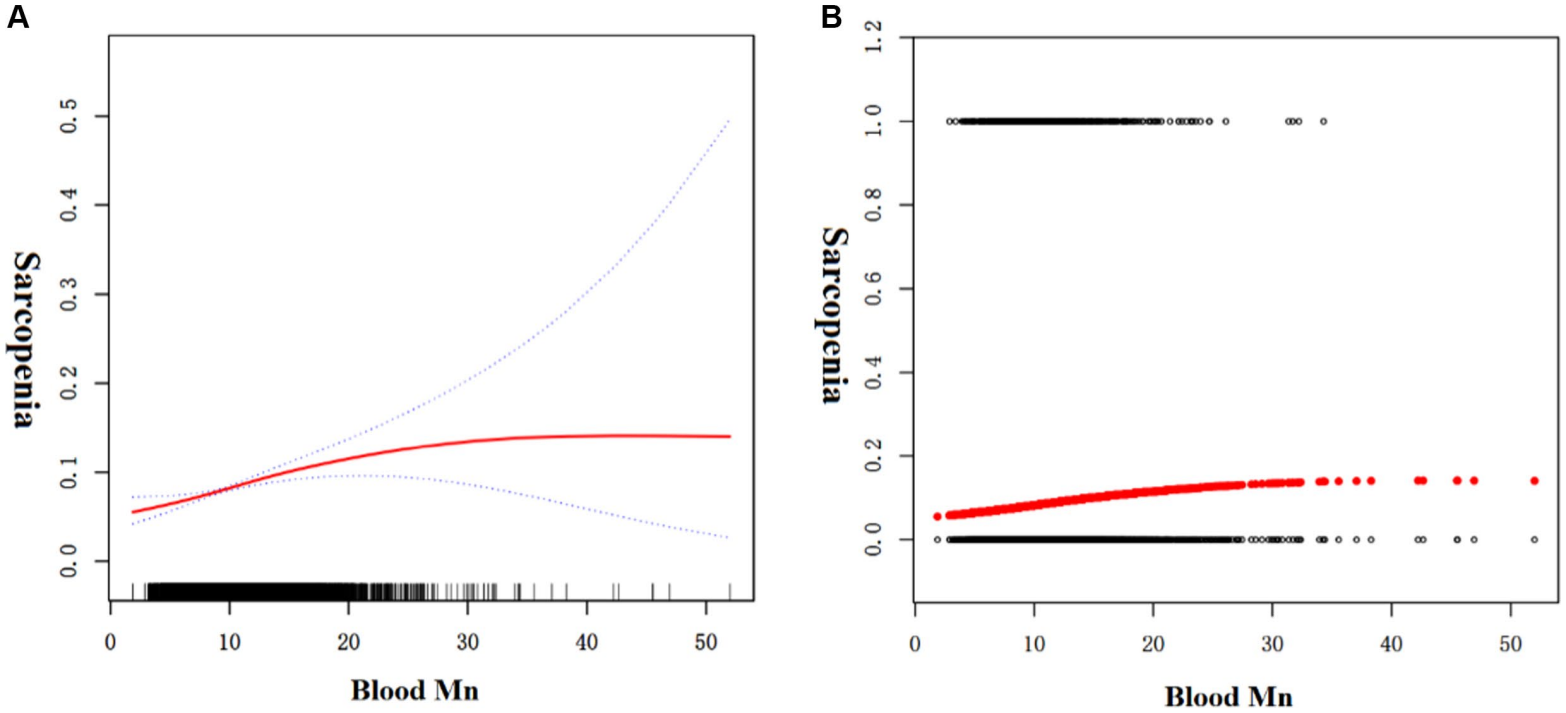
The association between blood Mn and sarcopenia. **(A)** Solid red line represents the smooth curve fit between variables. Blue bands represent the 95% of confidence interval from the fit. **(B)** Each black point represents a sample.

Since blood Mn levels are a continuous variable, analyzing for non-linear relationships is essential (16). In our study, depicted in Figure 1, we identified a non-linear correlation between blood Mn levels and sarcopenia after controlling for factors including age, gender, waist circumference, alcohol use, smoking status, diabetes, hypertension, cardiovascular conditions, educational attainment, BMI, and poverty index. Through the application of a two-piecewise linear regression model, we determined an inflection point to be 13.45 μg/L. To the right of the inflection point, the effect size was 1.006 with a 95% confidence interval (CI) of 0.996 to 1.048 and *p*-value of 0.769. Notably, a positive association between blood Mn levels and sarcopenia was evident on the left side of the inflection point, with an effect size of 1.082, a 95% CI of 1.043 to 1.122, and *p*-value of <0.001 (Table 4).

**Table 4:** The results of two-piecewise linear regression model.

Inflection point of blood Mn	Effect size (β)	95%CI	*p* value
<13.45 μg/L.	1.082	1.043, 1.122	<0.001
≥13.45 μg/L.	1.006	0.966, 1.048	0.769

Effect: sarcopenia, Cause: blood Mn. Adjusted: age, sex, waist circumference, alcohol consumption, smoking status, diabetes, hypertension cardiovascular, educational level, BMI, poverty level index.

### The results of subgroup analyses

As shown in Table 5, significant interactions were observed for gender and BMI (P for interaction <0.05). Conversely, for age, alcohol consumption, smoking status, physical activity levels, hypertension, and diabetes, the interactions were not statistically significant, with *p*-values above 0.05. Notably, the impact of blood Mn levels on sarcopenia differed significantly between genders and across various BMI groups. Blood Mn was positively associated with sarcopenia in male subjects [*β* = 1.075, 95%CI (1.041, 1.111)] and female subjects [*β* = 1.027, 95%CI (1.001, 1.1053)]. Blood Mn was positively associated with sarcopenia in different BMI groups [*β* = 1.093, 95%CI (1.049, 1.139); *β* = 1.067, 95%CI (1.027, 1.109); *β* = 1.021, 95%CI (0.995, 1.048), *p* = 0.021].

**Table 5:** Effect size of blood Mn levels on sarcopenia in prespecified and exploratory subgroups.

Characteristic	Effect size (95%CI)	P for interaction
Age		0.095
<40	1.059 (1.031, 1.087)	
≥40	1.024 (0.994, 1.056)	
Gender		0.029
Male	1.075 (1.041, 1.111)	
Female	1.027 (1.001, 1.053)	
BMI		0.021
<25	1.093 (1.049, 1.139)	
25–30	1.067 (1.027, 1.109)	
>30	1.021 (0.995, 1.048)	
Diabetes		0.431
Yes	1.023 (0.970, 1.079)	
No	1.046 (1.025, 1.069)	
Hypertension		0.393
Yes	1.030 (0.994, 1.068)	
No	1.049 (1.025, 1.074)	
Smoking status		0.583
Current	1.046 (1.021, 1.071)	
Former	1.020 (0.970, 1.072)	
Never smoker	1.055 (1.007, 1.105)	
Alcohol consumption		0.875
Non-drinker	1.042 (1.011, 1.073)	
1 to <5 drinks/month	1.062 (1.011, 1.115)	
5 to <10 drinks/month	1.032 (0.978, 1.089)	
10+ drinks/month	1.041 (1.004, 1.080)	
Physical activity		0.783
Vigorous	1.058 (1.013, 1.104)	
Moderate	1.040 (0.993, 1.088)	
None	1.040 (1.014, 1.066)	

## Discussion

In this study, we conducted an investigation into the potential relationship between blood Mn levels and the occurrence of sarcopenia in adults aged between 18 and 59 years, drawing on data from the National Health and Nutrition Examination Survey (NHANES) from 2011 to 2018. To the best of our knowledge, this represents the first population-based study that highlights a reverse U-shaped curve between blood Mn levels and sarcopenia, with an identified the inflection point at a blood Mn level of 13.45 μg/L. Our observations revealed disparate correlations between blood Mn levels and the presence of sarcopenia on the two sides of the inflection point (defined as blood Mn level = 13.45 μg/L). Our findings indicate a positive association between blood Mn levels and sarcopenia to the left of the inflection point, whereas to the right, the relationship did not reach statistical significance.

It is important to emphasize that the age range of participants in our study differs from that utilized in the pioneering work of Studenski et al. (14). Among the participants diagnosed with sarcopenia, comprising 8.2% of our study population, the average age is 43.05 ± 12.23 years. A significant 62.82% of the participants are aged 40 years or above. In contrast, individuals younger than 40 years comprise 37.18% of the study population. Landi et al. (17) suggest that the precursors of sarcopenia, including muscle loss and declining muscle function, can begin earlier in life. By extending our analysis to include adults under 40, we aim to explore these early indicators and potentially extend the understanding of sarcopenia’s onset and progression. The division at 40 years serves as a pragmatic threshold to investigate differential impacts of Mn exposure on muscle mass across varying life stages. Hughes et al. (18) suggest that significant changes in body composition, particularly in muscle mass and strength, may begin to manifest around this age, marking the onset of gradual sarcopenia development.

The association between blood Mn levels and sarcopenia is inherently complex due to manganese’s dual role as an essential nutrient and a potential toxic substance, with the degree of exposure playing a pivotal role in this complexity. Likewise, our research consistently points to the association between increased levels of Mn exposure and an elevated risk of sarcopenia, a pattern that was observed across all stratified subgroups, with the exception of gender and BMI. Sarcopenia, impacting almost one-third of the older person, represents a substantial factor in the development of negative health outcomes among geriatric patients (19, 20). The pathogenesis of sarcopenia encompasses a multitude of intricate cellular and molecular mechanisms, including neuromuscular junction dysfunction (21), a reduction in satellite cell number and function (22), a decline in the count of motor units (23), infiltration of intramuscular adipose tissue (24), proinflammatory processes (25), insulin resistance (25, 26)]. Moreover, mitochondrial dysfunction (27) and oxidative stress (28) are emerging as central elements in the pathogenesis of sarcopenia (27).

The underlying mechanisms of Mn toxicity are not fully determined, but mitochondrial dysfunction is a suspected contributor, as Mn accumulates in mitochondria and impairs their function, according to both *in vivo* and *in vitro* research (29, 30). Moreover, oxidative stress is linked to Mn toxicity, exacerbated when Mn undergoes a valence transition, amplifying its prooxidant capacity (31). Elevated levels of Mn within the mitochondria disrupt oxidative respiration, resulting in an overproduction of reactive oxygen species (ROS) and subsequent mitochondrial dysfunction (32). Given that Mn^3+^ possesses a more pronounced pro-oxidant potential compared to Mn^2+^, its formation within the mitochondria could exacerbate oxidative harm (33). This could impede the mitochondrial electron transfer chain, resulting in reduced ATP synthesis, increased electron escape, and elevated O_2_ production (34). Research by Remmen et al. (19) has shed light on the potential mechanisms underlying skeletal muscle weakness linked to oxidative stress during aging, specifically sarcopenia, through studies on mice deficient in CuZnSOD (Sod1KO), a cytoplasmic superoxide scavenger. These Sod1KO mice exhibit several phenotypes resembling characteristics of sarcopenia observed in aged wild-type (WT) mice, such as loss of innervation, mitochondrial dysfunction, and heightened production of mitochondrial ROS (mtROS) (10, 35, 36).

Mn accumulation could influence metabolic processes, particularly in relation to type 2 diabetes mellitus (T2M), which may subsequently contribute to the development and progression of sarcopenia. As a transition metal, manganese itself is an oxidant at high concentrations, and it appears to be involved in oxidative damage and mitochondrial dysfunction, which have been implicated in the development of T2D (37). Furthermore, T2DM has been identified as a significant risk factor for sarcopenia, likely due to increased inflammation, oxidative stress, and mitochondrial dysfunction associated with the disease (38, 39). Consequently, it is conceivable that Mn exposure could indirectly influence the development and progression of sarcopenia through its potential impact on metabolic status and the presence of T2DM.

Subgroup analysis plays a pivotal role in scientific research. In our study, we stratified data based on age, gender, BMI, diabetes, hypertension, smoking status, alcohol consumption, and physical activity. Notably, gender and BMI emerged as significant factors. Prior longitudinal studies (40, 41) have indicated that the decrease in height-adjusted muscle mass is less marked in women compared to men, potentially clarifying the gender-differentiated association between blood Mn levels and sarcopenia. However, the relationship between blood Mn levels and sarcopenia across various BMI groups remains unexplained, as it has not been reported in previous studies.

A significant strength of this study is its large and representative sample of the general, non-institutionalized population. We applied rigorous methods tailored for complex survey data, ensuring that our conclusions are applicable to the broader U.S. population. Our analysis was thorough, with adjustments for numerous confounders to solidify the reliability of our results. However, several limitations should be considered in our study. Firstly, the study’s cross-sectional design means exposures and outcomes were measured simultaneously, making it challenging to determine their temporal sequence. Thus, we cannot infer a cause-and-effect relationship, and the possibility of reverse causality remains. Furthermore, we used blood as the biomarker for manganese exposure, though alternative biological matrices, like hair, have been suggested as potentially more precise indicators. While we controlled for a wide array of potential confounders, there may be unaccounted factors such as occupational demands, family health history, and medication usage that could influence the findings, particularly when extrapolating to different populations. Additionally, it is pertinent to note that our study’s age range was also influenced by the inclusion criteria for Dual-energy X-ray Absorptiometry (DXA) measurements available in the NHANES database. The NHANES database restricts DXA measurements to individuals between the ages of 18 and 59 years. This exclusion could prevent the findings from being fully applicable to individuals beyond the age of 59, who might experience different health outcomes or levels of exposure to the studied factors. Finally, our focus was on the influence of individual metals on sarcopenia, without exploring the interactive effects that may result from exposure to multiple metals.

## Conclusion

Our study revealed a reverse U-shaped curve between blood Mn levels and sarcopenia, with an identified the inflection point at a blood Mn level of 13.45 μg/L. Additional studies are warranted to confirm our findings in prospective cohorts and to elucidate the potential mechanisms underlying the relationship between Mn and sarcopenia.

## Code availability

All analyses were performed with R software, V.4.1.3 [R: a language and statistical computing environment (program), Vienna, Austria: R Foundation for Statistical Computing, 2016], and EmpowerStats (http://www.empowerstats.com).

## Data availability statement

The original contributions presented in the study are included in the article/supplementary material, further inquiries can be directed to the corresponding author.

## Ethics statement

The studies involving humans were approved by the Institutional Review Board (or Ethics Committee) of the institutional review board of the National Center for Health Statistics, CDC (protocol #2005-06, #2011-17, #2018-01). The studies were conducted in accordance with the local legislation and institutional requirements. The participants provided their written informed consent to participate in this study.

## Author contributions

BX: Formal analysis, Funding acquisition, Supervision, Validation, Visualization, Writing – review & editing. Z-xC: Data curation, Investigation, Methodology, Software, Writing – original draft. W-jZ: Data curation, Methodology, Software, Supervision, Writing – original draft, Writing – review & editing. JS: Conceptualization, Data curation, Formal analysis, Investigation, Project administration, Resources, Software, Supervision, Visualization, Writing – original draft, Writing – review & editing. QZ: Conceptualization, Data curation, Formal analysis, Funding acquisition, Investigation, Methodology, Project administration, Resources, Software, Supervision, Validation, Visualization, Writing – original draft, Writing – review & editing.

## Footnotes

^1^ https://www.r-project.org

^2^ http://www.empowerstats.com
